# The trend of lead poisoning rate in Chinese population aged 0–18 years old: a meta-analysis

**DOI:** 10.1186/s12889-015-2103-9

**Published:** 2015-08-06

**Authors:** Min-ming Li, Jia Cao, Zhen-yan Gao, Xiao-ming Shen, Chong-huai Yan

**Affiliations:** MOE–Shanghai Key Laboratory of Children’s Environmental Health, Xinhua Hospital, Shanghai Jiao Tong University School of Medicine, Shanghai, 200092 PR China

## Abstract

**Background:**

Childhood lead poisoning is a public health problem gained widely attention for the health damage caused by lead exposure. Pediatrics defines lead poisoning as BLL of or higher than 10 μg/dL, which leads to harmful effects in nervous system, hematological system and urinary system. This study investigates the percentage of 0–18 year old Chinese population with blood lead level (BLL) ≥10 μg/dL during 1990–2012 by searching epidemiologic studies from electronic database focused on BLL in mainland China.

**Methods:**

Epidemiologic studies about BLL in China mainland between January 1990 and October 2012 were searched from electronic databases including CNKI, CBM disc, Wanfang Data, Pubmed and Medline. Data extraction, data analysis and risk of bias assessments were performed.

**Results:**

Fifty-five articles were included in analysis containing 200,002 subjects, covering 19 provinces, autonomous regions and municipalities. The corrected pooled rate by trim and fill method under random effect model was 9 % (95 CI: 6 %, 12 %). The corrected pooled lead poisoning rate by trim and fill method was 28.1 % (95 % CI: 21.6 %, 34.6 %) from data published during 1990–2000, much higher than the rate during 2001–2005 (10.5 %, 95 % CI: 6.4 %, 14.5 %) and the rate during 2006–2012 (5.3 %, 95 % CI: 3.7 %, 7 %). The corrected pooled lead poisoning percentage in eastern zone (4.3 %, 95 % CI: 2 %, 6.6 %) was slightly lower than that in western zone (5.8 %, 95 % CI: 3.2 %, 8.5 %) and much lower than in central zone (8.5 %, 95 % CI: 4.9 %, 12.1 %). The corrected pooled rate of population living around mining area (70 %, 95 % CI: 62.7 %, 77.3 %) was much higher than that of population in urban area (9.6 %, 95 % CI: 7.1 %, 12.1 %), suburban area (23.6 %, 95 % CI: 17 %, 30.3 %), rural area (23.8 %, 95 % CI: 6.7 %, 40.9 %) and industrial area (57.5 %, 95 % CI: 28 %, 86.9 %). In male population, the corrected pooled rate (10 %, 95 % CI: 7 %, 13 %) was slightly higher than that in female population (7.7 %, 95 % CI: 5 %, 10.4 %). Considering different age groups, the lead poisoning prevalence gradually rose with the increase of age and reached peak level at preschool age, then declined slightly with age.

**Conclusion:**

This meta-analysis revealed lead exposure situation of Chinese population in recent decades which provide robust evidence for policy making.

## Background

Lead is a toxic heavy metal and can cause damage to human organs and tissues, particularly to the development of central nervous system. Lead exposure in children might cause permanent learning and behavior disorders [1, 2]. Diagnosis of lead exposure is based on blood lead level (BLL) [3]. The American Academy of Pediatrics defines lead poisoning as BLL of or higher than 10 μg/dL [4]. Thus, the U.S. Centers for Disease Control and Prevention and World Health Organization stated that a BLL of 10 μg/dL or above needs to be concerned. However, lead exposure may have harmful effects on multiple organs even at lower levels [5, 6].

Data from National Health and Nutrition Examination Survey (NHANES) in USA indicated that an estimated percentage of BLL ≥10 μg/dL in children aged 1–5 years was 88 % during 1976–1980, then fell sharply to 4.4 % during 1991–1994, to 1.6 % during 1999–2002, and to 0.8 % during 2007–2010 [7–10]. In developed countries, children living in poor families with lower educational level are at higher risk of elevated BLL [8–11]. The situation is similar in the developing countries. Among Central and South American children, the percentage of children with BLL ≥10 μg/dL is 33 ~ 34 % [12]. The world disease burden from lead poisoning in Western Pacific accounts for about one fifth, and in Southeast Asia accounts for another fifth [12].

The Chinese government has begun to pay attention to childhood lead poisoning since 1990s, and at mean time Chinese scholars have conducted a couple of epidemiological studies to investigate childhood lead poisoning in local areas of China. To provide convincing evidence for policy making regarding children lead poisoning prevention, this study investigated the national trend of lead poisoning rate in the 0–18 year old of Chinese population during 1990–2012 by analyzing the published data.

## Methods

### Literature searching strategy

We searched epidemiologic studies about BLL in China mainland from electronic databases, CNKI, CBM disc, Wanfang Data, Pubmed and Medline between January 1990 and October 2012. The searching strategy was “blood lead level” OR “lead poisoning” in Chinese and English language. Two authors firstly screened articles by reading the titles and abstracts, and then reviewed the full text of the eligible publications.

### Literature quality assessment

The assessing criteria based on the standard recommended by Agency for Healthcare Research and Quality (AHRQ) containing nine items was used to evaluate literature quality [13]. The principals of literature quality assessment were described in Table 1. Two authors evaluated each eligible article independently. The discrepancy between two authors was solved through discussion.

**Table 1:** Assessment of observing study quality based on the standard recommended by AHRQ

Assessing principal^a^	
Study question clearly focused	
Sampling of Study Population (Random, Convenient, Self-selected)	
Clear definition of including or excluding criteria	
Clear definition of research time	
Assessment of confounding effects of various factors (for example, age of the patients, patient sex)	
Appropriate measure of precision	
Clear description of response rate	
Clear description of dealing with missing data	
Conclusions supported by results with possible bias and limitations taken into consideration	

^a^scoring method: yes = 1; no or not mentioned = 0

### Inclusion criteria

1. Study subjects were not the specific lead exposure population; 2. Sample size should be not less than 100 (for neonatal, not less than 50); 3. BLL detection was under strict quality control; 4. Results must comprise the number of the subjects and number of lead poisoning subjects. Lead poisoning is defined as BLL ≥ 10 μg/dL in the studies included.

### Data extraction

Two authors did data entry independently and cross-checked. The following details were recorded: authors, title, study year, study site, sample type, testing method, sample size, age range, gender, number of the subjects and number of lead poisoning subjects.

### Data analysis

Heterogeneity was assessed by Cochran chi-square (*χ*^2^) and quantified with the I^2^ statistic, which was low when I^2^ was less than 25 %, moderate when between 25 % ~ 50 %, high when more than 50 % [14]. Considering heterogeneity across studies, a random-effects model was used to calculate pooled lead poisoning rate and 95 % confidence intervals (CIs). DerSimonian and Laird method might be a robust approach to combine weighted effect size under random-effects model. Subgroup analysis was used to deal with heterogeneity. Publication bias was evaluated by Begg’s Test and Egger’s test [15]. Significance was set at a *P* value less than 0.05. Stata version 12.0 (StataCorp, College Station, Texas, USA) was used for all statistical calculations.

## Results

### Searching result

**Fig. 1:**
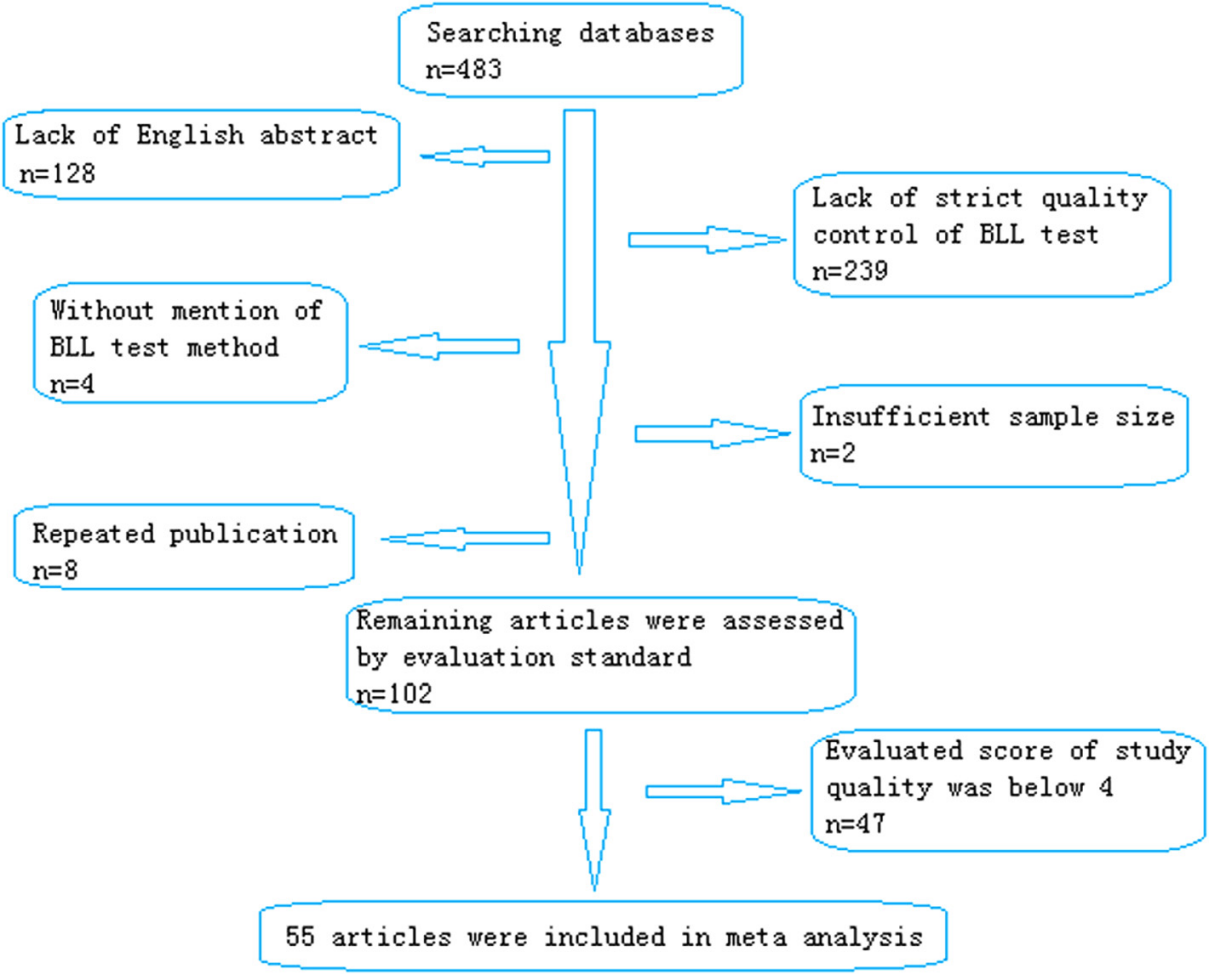
Flow diagram of the study selection process

Four hundred eighty-three articles were retrieved after searching the medical databases illustrated above. One hundred twenty-eight articles were excluded due to no English abstract. Two hundred thirty-nine articles were excluded due to lack of strict quality control of BLL test method. Atomic absorption spectrometry (AAS), inductively coupled plasma mass spectrometry (ICP-MS) and anodic stripping voltammetry (ASV) were acceptable BLL test methods in this study. Strict quality control (QC) should be performed in sample collection to avoid contamination. A daily QC procedure of library should be performed and at least two controls of low (<10 ug/dL) and high lead (>20 ug/dL) levels should be tested to ensure the validity of test results. The acceptable range for detecting accuracy should not be greater than ±4 ug/dL or ±10 %. For testing precision, the relative standard deviation (RSD) should not be more than 15 % when blood lead level between 2 to 10 ug/dL, and not be more than 10 % when level above 10 ug/dL. The libraries were participated in the external quality assessment (EQA) of the national center for clinical laboratory. Four articles did not mention BLL test method. Eight articles were repeated publication. Two articles were ineligible due to insufficient sample size. The remaining 102 articles were assessed of study quality by evaluation standard, which was recommended by Agency for Healthcare Research and Quality (AHRQ) [13]. Mean evaluated score of study quality was about four. The 55 articles above or equal to four scores were included in this study (Fig. 1, Table 2). All the included studies contained 200,002 subjects, covering 19 provinces, autonomous regions and municipalities. The characteristics of included studies were showed in Table 3 [16–70].

**Table 2:** Characteristics of included studies, by researching regions

	Eastern zone^18–50^	Central zone^51–56^	Western zone^57–68^	National wide^69–72^
Number of studies	33	6	12	4
Published year				
1990–2000	9	1	5	0
2001–2005	15	4	3	4
2006–2012	9	1	4	0
Sample size				
<500	8	2	7	0
500–1499	15	3	3	0
1500–2999	8	1	1	0
≥3000	2	0	1	4

**Table 3:** Information and data extracted from 55 publications in China

Survey time	Sites	Testing method^a^	Sample size	Districs (Sample size)	Age range
2010	Guangdong province	1	1517		0–14 years
2003	Foshan	2	5175		3–6 years
2003	Henan province	2	527	urban (251)	3–5 years
				industrial (276)	
2003	Foshan	2	588	urban (346)	newborn
				suburban (242)	
2006	Nanjing	1	404	urban (404)	9–12 years
2008	Ya’an	1	703		0–12 years
2001	Henan province	1	585		0–6 years
2005	Tai’an	1	1200		7–12 years
2004	Xuzhou	1	1070		0–6 years
1997	Guangxi province	1	1508	urban (1508)	newborn
1993	Shanghai	1	348	urban (348)	newborn
1997	Zhejiang province	1	1320	Urban (1024)	0–6 years
				suburban (296)	
1997	Beijing	1	270	urban (270)	0–18 months
2006	Hohhot	1	585		7–12 years
2005	Urumqi	1	987	urban (987)	6–10 years
2004	linyi	1	1228	urban (1228)	2–6 years
2004	Guangzhou	1	1215	urban (1215)	0–6 years
2008	Nanjing	1	1258	urban (1258)	3–6 years
2004–2006	China	1	44,045	urban (44,045)	0–6 years
2005	China	1	17,141	urban (17,141)	0–6 years
2010	Guangzhou	1	2464	urban (2464)	0–6 years
2005	Lishui	1	2369	urban (2369)	3–6 years
2006	Anshan	1	408	urban (408)	3–6 years
2002	Foshan	2	152		newborn
2002	Guangzhou	2	653	urban (653)	0–6 years
2003	Wuhu	2	300	urban (193)	3–5 years
				suburban (47)	
				industrial (56)	
2002	Guangzhou	2	1905	urban (1518)	3–14 years
				rural (387)	
2002	Anshan	1	913	urban (150)	0–6 years
				suburban (150)	
				industrial (300)	
				mining (150)	
2001	Beijing	1	2262		0–6 years
2004	Jinchang	1	256		2–7 years
2002	Lanzhou	1	262		0–3 years
1998	Lanzhou	1	103	urban (103)	newborn
2000	Urumqi	1	138		newborn
2001	Taiyuan	1	395		4–6 years
1998	Lanzhou	1	160		3–6 years
1997	Wuxi	1	1249		0–5 years
2010	Nanjing	1	1113	urban (1113)	7–12 years
2010	Hunan province	1	2044	Urban (2044)	2–6 years
2011	Kunming	1	100		newborn
2004	Zhejiang province	1	240		3–6 years
2008	Shanghai	1	1652		newborn
2007	Guangzhou and Shenzhen	2	761	urban (430)	2–12 years
				suburban (257)	
				rural (74)	
2011	Guangzhou	2	1570		6–14 years
2000	Guiyang	1	366		9–11 years
2004	Jintan	1	1344	urban (933)	3–5 years
				suburban (187)	
				rural (150)	
1999	Ningbo	1	1018	urban (1018)	2–6 years
1997–1999	Shanghai	1	5933		1–6 years
2008	Chengdu	1	4436		0–7 years
2004–2008	China	1	69,968	urban (69,968)	0–6 years
2002	China	2	6502	urban (6502)	3–5 years
1997	Wuxi	1	1117	urban (589)	1–5 years
				suburban (528)	
2004–2008	Beijing	1	5018	urban (5018)	0–6 years
1993	Shanghai	1	132	urban (132)	newborn
1993	Beijing	1	141	urban (141)	newborn
2000	Nanchang	1	883	urban (883)	2–8 years

^a^1-AAS;2-ICPMS

### Pooled analysis of lead poisoning rate in Chinese population from data published during 1990–2012

**Fig. 2:**
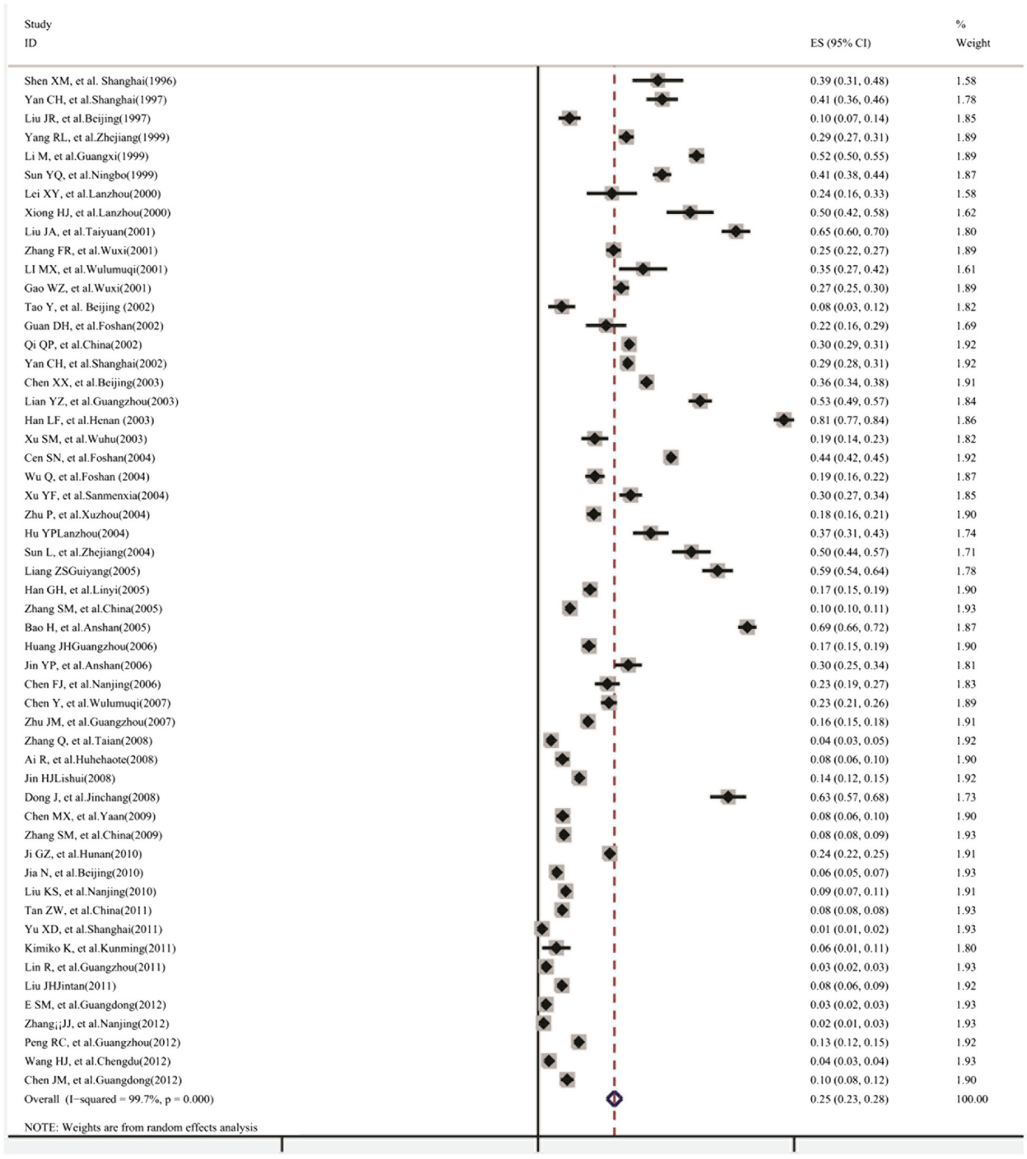
Forest plot of pooled analysis of lead poisoning rate in the 0–18 year old Chinese population during 1990–2012

Fifty-five studies were included in pooled analysis of unadjusted lead poisoning rate. Homogeneity test showed high heterogeneity between studies (*χ*^2^ = 17151.45, *p* = 0.000, I^2^ = 99.7 %). Under random effect-model, combined lead poisoning rate in the 0–18 year old Chinese population from data published during 1990–2012 was 25 % (95 % CI: 23 %, 28 %) (Fig. 2). Begg’s Test and Egger’s test showed the existence of publication bias (*p* < 0.05). Trim and fill method was used to provide an estimate of the number of missing studies and adjusted the pooled effect size for the publication bias [71]. In this article, 24 studies with the lead poisoning rates from 10 to 66 % were filled and used in the following analyses based on Trim and fill method. An adjusted intervention effect under random effect model was 9 % (95 CI: 6 %, 12 %) when 24 studies were supplemented.

The high heterogeneity might be associated with discrepancy of time, study sites, gender and age ranges of included studies. To deal with heterogeneity, subgroup analysis was used which was based on study year ranges, different economic zones, gender and different age ranges. Table 4 described pooled lead poisoning rates which corrected by trim and fill method due to publication bias in different subgroups, such as different published year ranges, economic zones, gender and age ranges groups respectively.

**Table 4:** Lead poisoning rate according to published year, different economic zones, gender and different age ranges

Subgroup	Pooled rate (95 % CI) (%)	Sample size	Heterogeneity test	P	I^2^
Published year					
1990–2000	28.1 % (21.6 %, 34.6 %)	13,804	845.98	<0.05	98.2 %
2001–2005	10.5 % (6.4 %, 14.5 %)	111,663	9525	<0.05	99.7 %
2006–2012	5.3 % (3.7 %, 7 %)	73,629	1560.91	<0.05	98.7 %
Economical zones					
Eastern zone	4.3 % (2 %, 6.6 %)	99,896	14819.93	<0.05	99.5 %
Central zone	8.5 % (4.8 %, 12.1 %)	50,543	4572.20	<0.05	99.3 %
Western zone	5.8 % (3.2 %, 8.5 %)	48,393	3956.12	<0.05	99.1 %
Districs					
Urban area	9.6 % (7.1 %, 12.1 %)	167,058	6980.4	<0.05	99.5 %
Suburban area	23.6 % (17 %, 30.3 %)	2555	114.22	<0.05	93.9 %
Rural area	23.8 % (6.7 %, 40.9 %)	1416	184.99	<0.05	98.4 %
Industrial area	57.5 % (28 %, 86.9 %)	937	403.16	<0.05	99.3 %
Mining area	70 %^a^ (62.7 %, 77.3 %)	150	0	<0.05	
Sex					
Male	10 % (7 %, 13 %)	100,036	3676.19	<0.05	99.4 %
Female	7.7 % (5 %, 10.4 %)	82,754	2947.68	<0.05	99.2 %
Age range					
Newborn (born-1 month)	3.3 % (0, 14.8 %)	4978	1970.40	<0.05	99.5 %
Infant(1 month–1 year old)	4.7 % (2.8 %, 6.7 %)	11,551	125.12	<0.05	90.4 %
Toddler (1–3 years old)	7.4 % (4.8 %, 10 %)	28,324	1532.99	<0.05	97.7 %
Pre-school age (3–6 year old)	9.5 % (7.8 %, 11.2 %)	99,735	3623.39	<0.05	98.2 %
school age (6–12 years old)	6.7 % (4.7 %, 8.6 %)	20,873	825.25	<0.05	95.6 %
Adolescence (>12 years old)	7 % (0, 13 %)	435	17.99	<0.05	88.9 %

^a^The data is a constant for only one literature included

#### The factor of time

**Fig. 3:**
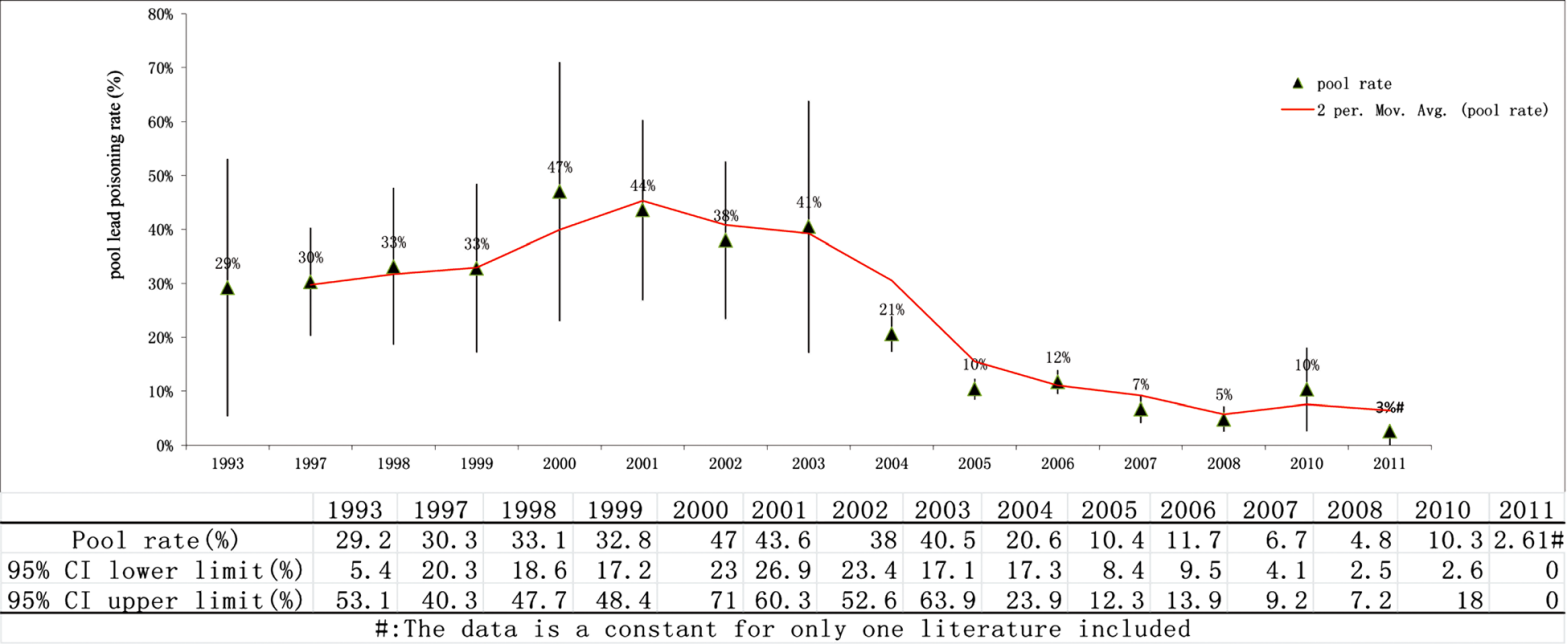
The temporal trend of lead poisoning rate in the 0–18 year old Chinese population during 1990–2012

The corrected pooled lead poisoning rate by trim and fill method was 28.1 % (95 % CI: 21.6 %, 34.6 %) from data published during 1990–2000, much higher than the rate during 2001–2005 (10.5 %, 95 % CI: 6.4 %, 14.5 %) and the rate during 2006–2012 (5.3 %, 95 % CI: 3.7 %, 7 %). Figure 3 showed an overall decreasing temporal trend of lead poisoning rate with study year through analysis tool of moving average line. The tendency of lead poisoning rate showed that the rate increased gradually in the 1990s, and dropped slowly during 2000–2003, then decreased rapidly after 2003.

#### The factor of different economic zones

**Fig. 4:**
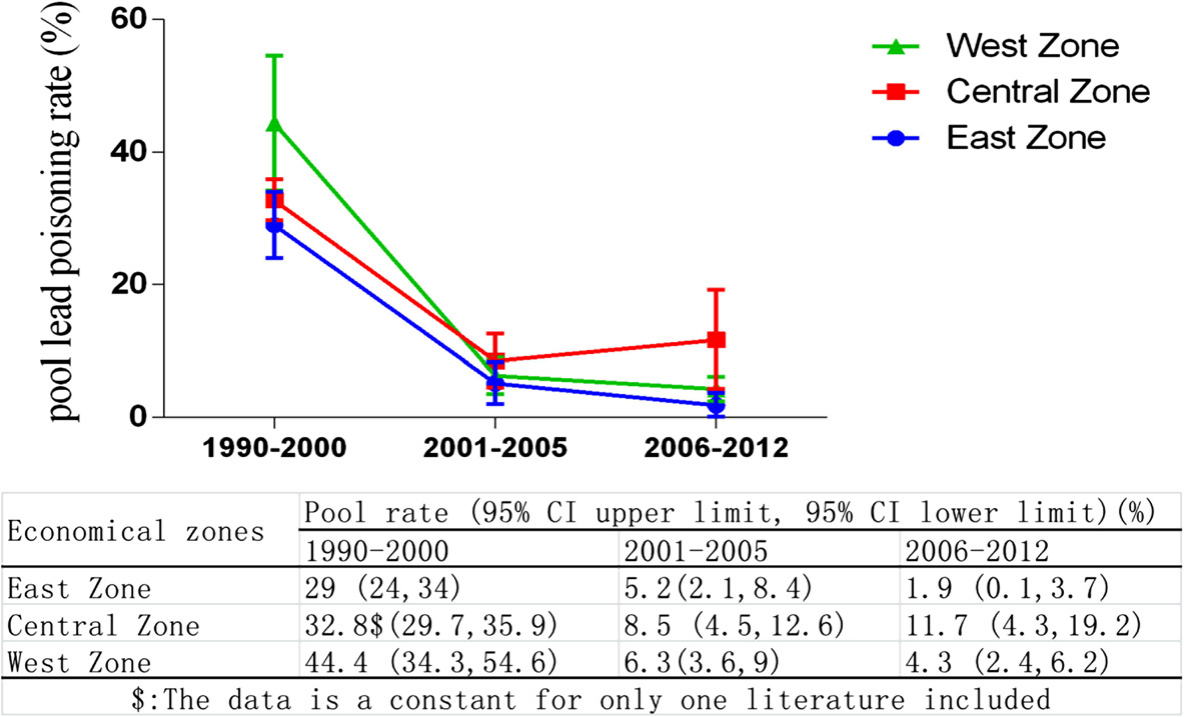
The temporal trend of lead poisoning rates among three economic zones in China main land

According to economic level and geographic area, the provinces, autonomous regions and municipalities in mainland China are categorized into three zones: eastern, central and western. *1*). The eastern zone includes Beijing, Tianjin, Hebei, Shanghai, Jiangsu, Zhejiang, Fujian, Shandong, Guangdong, Hainan and Liaoning. *2*). The central zone includes Shanxi, Anhui, Jiangxi, Henan, Hubei, Hunan, Jilin and Heilongjiang. *3*). The western zone includes Inner Mongolia, Guangxi, Chongqing, Sichuan, Guizhou, Yunan, Tibet, Shanxxi, Gansu, Qinghai, Ningxia and Xinjiang [72]. The corrected pooled lead poisoning percentage in eastern zone (4.3 %, 95 % CI: 2 %, 6.6 %) was slightly lower than that in western zone (5.8 %, 95 % CI: 3.2 %, 8.5 %) and much lower than in central zone (8.5 %, 95 % CI: 4.9 %, 12.1 %). An interact effect between economic zones and study year ranges on rate of BLL ≥ 10 μg/dL was noted when analyzing variance model with weighed least squares method (WLS) by sample size of included studies was applied (*p* < 0.05). When considering the effect of economic level on rate of BLL ≥ 10 μg/dL, all temporal tendencies of the rate were decreasing among three economic zones as showed in Fig. 4. But it should be highlighted that the relatively slower declining tendency in central zone compared to those in western zone and eastern zone.

#### The factor of living districts

**Fig. 5:**
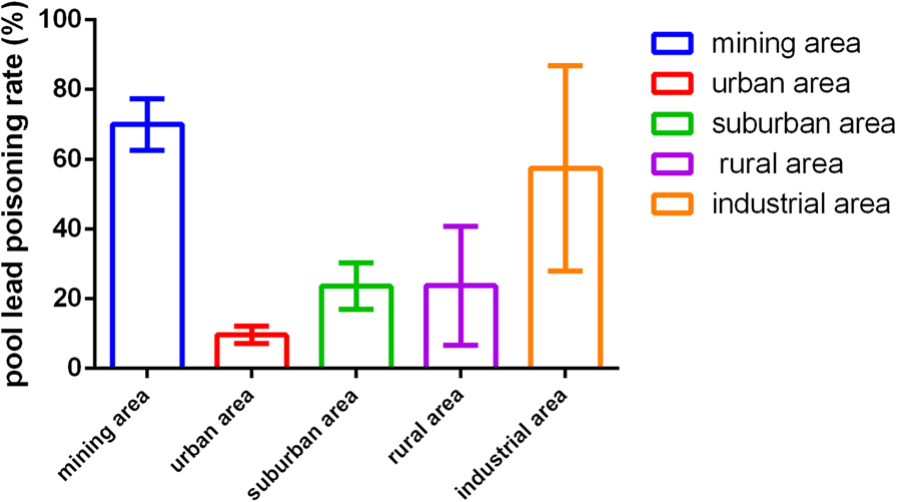
The pooled lead poisoning rates among different districts

Only one literature reported that the lead poisoning rate among population living around mining area was 70 % (95 % CI: 62.7 %, 77.3 %) which was much higher than the corrected pooled rates in urban area (9.6 %, 95 % CI: 7.1 %, 12.1 %), suburban area (23.6 %, 95 % CI: 17 %, 30.3 %), rural area (23.8 %, 95 % CI: 6.7 %, 40.9 %) and industrial area (57.5 %, 95 % CI: 28 %, 86.9 %) from data published during 1990–2000. Figure 5 showed lead poisoning rates among different districts.

#### The factor of gender

Gender information was available in 21 studies. The corrected pooled lead poisoning rate by trim and fill method of male population aged 0–18 year old in China (10 %, 95 % CI: 7 %, 13 %) was slightly higher than that of female (7.7 %, 95 % CI: 5 %, 10.4 %).

#### The factor of age range

**Fig. 6:**
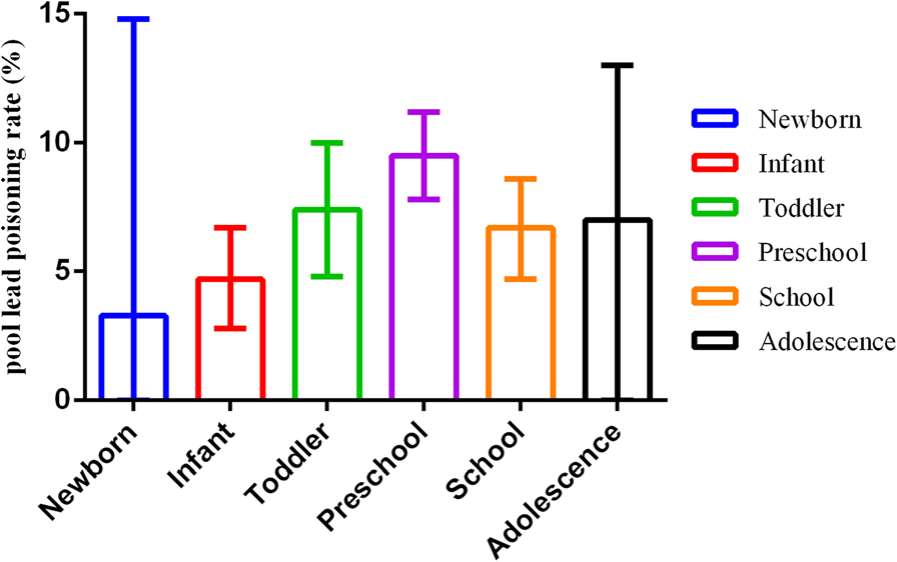
The pooled lead poisoning rate with different age ranges

**Fig. 7:**
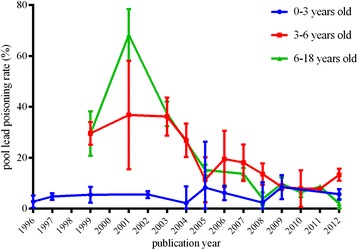
The pooled lead poisoning rate in different age group over the years

The corrected lead poisoning rate by trim and fill method of preschool children between 3 to 6 years old (9.5 %, 95 % CI: 7.8 %, 11.2 %) was higher than that of other age ranges including newborn (3.3 %, 95 % CI: 0 %, 14.8 %), infant (4.7 %, 95 % CI: 2.8 %, 6.7 %), toddler under 3 years old (7.4 %, 95 % CI: 4.8 %, 10 %), school age between 6 to 12 years old (6.7 %, 95 % CI: 4.7 %, 8.6 %) and adolescence (7 %, 95 % CI: 0, 13 %). The lead poisoning rate of population aged 0–18 year old indicated lead exposure level. Figure 6 presented that the lead poisoning rate gradually rose with the increase of age and reached peak level at preschool age, then declined slightly with age. The temporal trends of lead poisoning rate in different age groups showed that the downward trend of prevalence with year was more obvious in population above 3 years old compared to population under 3 years old (Fig. 7).

## Discussion

With increasing number of epidemiological studies about BLL in China in recent years, it is now possible to obtain direct evidence of lead poisoning situation of Chinese population aged 0–18 years old. Based on strict inclusion criteria, eliminated relative low-quality studies by quality assessment of cross-sectional study, we identified 55 articles in this meta-analysis including 200,002 subjects, covering 19 provinces, autonomous regions and municipalities, thus the large population guaranteed the reliability of this study.

In this study, pooled rate of BLL ≥10 μg/dL in the 0–18 year old Chinese population during 1990–2012 was 25.1 % and corrected pooled rate under trim and fill method was 9 %, both much higher than all levels during 1991–1994 (4.4 %), 1999–2002 (1.6 %) and 2007–2010 (0.8 %) in US [8–10].

Considering high heterogeneity, subgroup analysis of different study year ranges showed overall decreasing trend of the rate with time. The decline appears to be attributed to government efforts of controlling lead pollution, including the phasing out of leaded gasoline, converting household fuel from coal to natural gas or other clean energy alternatives, and shutting down heavily polluted enterprises. But this study revealed that during 1993–2000 of survey time when leaded gasoline was not prohibited in China mainland and China had been undergoing rapid and drastic industrial development, tendency of lead poisoning rate was slightly increasing with time [43]. After early 21 century when leaded gasoline was gradually phased out in China mainland, the results showed steadily decreasing trend of the rate with time [73]. This result was consistent with studies conducted in USA which revealed the close correlation between the decreasing use of gasoline with lead and sharply declining BLL simultaneously [74, 75]. In the age of leaded gasoline, the lead dust mainly attributed to the exhaust of leaded gasoline and diesel fuel, while the lead-free gasoline brought down the atmosphere lead concentration [76–78].

Although the Chinese government and researchers continued to pay efforts to lead poisoning control and take preventative measurements for several decades, the study found that the declining tendency of lead poisoning rate was slow in the short term during 2000–2003. Several factors contributed to that as below. Though atmospheric lead emission from vehicle exhaust was decreasing rapidly in short period of time after unleaded gasoline application in China, it was hard to eliminate the lead accumulated in the soil in short time, which caused environmental pollution and potential lead exposure to high risk population, such as children and women [79, 80]. After 2003 of survey time, the rate was decreasing rapidly from about 40 to 2.61 % in 2011. Although the rate of BLL ≥10 μg/dL gradually declined with time, in this study pooled rate was 8 % from data published during 2006–2012, still much higher than that (0.8 %) observed in the surveys conducted in USA from 2007 to 2010 [10]. One of the key factors contributed to this observation in China might be the increasing lead discharge from the industrial emission [81]. The data showed that coal consumption, as the most important contributing factor of the lead discharge, continued to rise in recent decades in China [81].

When considering the factor of economic level, we found that pooled rate of BLL ≥10 μg/dL in eastern zone was slightly higher than that in the other two zones and still presented a sharp decreasing tendency since 2000. It is well known that low family income was a statistically significant independent predictor of elevated children’s BLL [8]. According to the data of Chinese statistical inspection, the annual per-capita income of people in eastern China was much higher than that in western China [82]. However, the slightly increasing tendency of lead poisoning rate in central zone since 2000 should be explained cautiously for a small quantity of epidemiological data. During 2006–2010, only five studies were included in this meta-analysis and rate of BLL ≥10 μg/dL in Hunan province was 24 % which accounted for a relatively large weight in pooled analysis [52].

Pooled lead poisoning rate of male population aged 0–18 years old (24.9 %) was slightly higher than that of female, which was similar to what was observed in the third National Health and Nutrition Examination Surveys (NHANES III) during 1999–2004 in USA [8]. It might be associated with more outdoor activity for male population, which leads to higher risk of lead exposure. As for age factor, the lead poisoning percentage peaked at pre-school age (3–6 year old) and then declined with age, similar to the result of analogical study in Chinese population, but not consistent with the result of previous studies in which the percentage of BLL ≥10 μg/dL was higher in children aged 1 to 2 years old than 3–5 years old, which needs to be explored further [8, 82].

There were several limitations needed to be classified: (a) for the coverage of evidence base, adequate epidemiological surveys encompassing the geographical, racial, economic, cultural, social diversity of nation as a whole were required. In this meta-analysis, the number of national wide surveys was small and data was missing during the periods of 1990–2000 and 2006–2012. The data from poor rural areas was limited, and this might cause underestimation of the pooled rate; (b) homogeneity test showed high heterogeneity between studies. The heterogeneity was still high within subgroups, which might be influenced by other factors, such as geographical distribution, living environment, living habits and ethnicities [83, 84]; (c) in this meta-analysis, publication bias still exists, which might be caused by investigator’s loss of interest in the topics with null (negative or inconclusive) results, editor’s preference to articles with positive results, etc. [85, 86]. The publication bias in this study might lead to overestimation of the rate of BLL ≥10 μg/dL. Thus, trim and fill method should be applied to correct the pooled effect size by supplementing 24 studies [71].

## Conclusions

In conclusion, the overall rate of BLL ≥10 μg/dL in Chinese population aged 0–18 years old during 1990–2012 presented persistent declining trend along with time. Pooled percentage was slightly lower in eastern zone than other two economic zones. The rate was slightly higher in male population than in female population. The lead poisoning rate of Chinese population during postnatal period climbed from infancy, peaked at pre-school age and then declined with age. This meta-analysis revealed lead exposure situation of Chinese population in recent decades which provide robust evidence for policy making.

## Abbreviation

CI: Confidence interval
BLL: Blood lead level
NHANES: National Health and Nutrition Examination Survey
AHRQ: Agency for Healthcare Research and Quality
WLS: Weighed least squares
AAS: Atomic absorption spectrometry
ICPMS: Inductively coupled plasma mass spectrometry
